# Asymptomatic Brain Edema after Hemodialysis Initiation in a Patient with Severe Uremia

**DOI:** 10.1155/2017/9265315

**Published:** 2017-05-03

**Authors:** Kiichiro Fujisaki, Kaneyasu Nakagawa, Hiroshi Nagae, Toshiaki Nakano, Masatomo Taniguchi, Kosuke Masutani, Takanari Kitazono, Kazuhiko Tsuruya

**Affiliations:** ^1^Department of Medicine and Clinical Science, Graduate School of Medical Sciences, Kyushu University, Fukuoka, Japan; ^2^Department of Integrated Therapy for Chronic Kidney Disease, Graduate School of Medical Sciences, Kyushu University, Fukuoka, Japan

## Abstract

A 66-year-old man with severe renal insufficiency presented with mild confusion associated with uremia. Cranial magnetic resonance imaging (MRI) showed no remarkable changes. The patient was placed on short-duration hemodialysis (2 hours) with smaller surface area and low blood flow (100 mL/min) to avoid dialysis disequilibrium syndrome (DDS). His consciousness gradually improved and he did not develop apparent DDS symptoms. However, T2-weighted FLAIR MRI showed increased signal intensities bilaterally in the cortical and subcortical areas of the occipital lobe on day 15. In other words, cranial MRI showed cerebral edema, indicating asymptomatic DDS. On day 29, cranial MRI showed a return to findings on admission. In this case, because the patient did not have apparent DDS symptoms despite MRI changes, we diagnosed asymptomatic cerebral edema. The patient was discharged on regular intermittent HD without any neurological deficits. No further neurological disturbances were noted during 1-year follow-up. MRI findings in ESKD patients without DDS symptoms help to clarify the diagnosis of cerebral edema. In this case, the patient did not have apparent DDS symptoms and was therefore diagnosed with asymptomatic cerebral edema.

## 1. Introduction

Uremic complications in the central nervous system occur in both acute kidney injury and end-stage kidney disease (ESKD). Uremic encephalopathy is likely caused by multiple metabolic derangements but its precise pathophysiology remains unknown. The major clinical features include alterations in mental function and level of consciousness, myoclonus, tremor, and focal or generalized seizures.

Dialysis disequilibrium syndrome (DDS), first described by Kennedy et al. [1], has been recognized for more than 50 years in those undergoing dialysis treatments. DDS is a clinical syndrome of neurologic deterioration generally seen during initial treatment sessions in patients undergoing hemodialysis (HD) [2–4]. DDS typically presents with coma, seizures, headache, and nausea. The precise incidence of DDS is not known but seems to be decreasing, most likely because patients today begin dialysis at lower urea concentrations than previously [4].

Previous studies have reported cerebral edema in patients with DDS, based on autopsy data and brain imaging [5, 6]. Galons et al. reported that magnetic resonance imaging (MRI) confirmed the presence of cerebral edema in nephrectomized rats receiving HD [7]. Based on diffusion-weighted MRI, these authors reported that the cerebral edema was interstitial and not intracellular [7].

We report here a severely uremic patient with cerebral edema secondary to hemodialysis and the subsequent regression of cerebral edema.

## 2. Case Report

A 66-year-old man was transferred to our hospital because of confusion, severe general fatigue, and dyspnea of 1-week duration. The patient had chronic kidney disease related to diabetic nephropathy. Although we strongly recommended initiation of chronic HD, the patient refused the treatment.

The patient had a 15-year history of type 2 diabetes mellitus with triopathy. Other medical history included hypertension of unknown duration that was being treated with amlodipine besylate, losartan, and carvedilol. There was no prior history of seizures, neurological symptoms, loss of vision or other underlying illnesses, or use of any other drugs. Upon arrival at our outpatient department, the patient had severe dyspnea.

On admission (day 0), the patient had mild confusion and anasarca. His height was 168 cm; body weight, 74 kg; blood pressure, 124/43 mmHg; and body temperature, 35.7°C. Urinalysis showed 1+ proteinuria and 2+ occult blood, − glucose, and − ketone and urinary sediment revealed 1–4 erythrocytes, 1–4 leukocytes, 1–4 squamous cells, and 0-1 transitional cells per HPF, but no casts. Blood tests showed low hemoglobin (4.6 g/dL), white blood cell count of 5240/*µ*L (82.3% neutrophils, 12.8% lymphocytes, 1.1% eosinophils, 3.6% monocytes, and 0.2% basophils), and thrombocytopenia (platelet count 84 × 10^3^/*µ*L). Blood chemistry showed blood urea nitrogen (BUN) of 222 mg/dL; serum creatinine, 25.4 mg/dL; total protein, 5.7 g/dL; albumin, 3.3 g/dL; lactate dehydrogenase, 403 IU/L; aspartate aminotransferase, 46 IU/L; alanine aminotransferase, 44 IU/L; serum sodium, 139 mmol/L; and serum potassium, 6.1 mmol/L, glucose 97 mg/dL. Serological tests showed C-reactive protein of 0.2 mg/dL. Coagulation parameters were normal. Chest X-ray showed congestion of the lung fields. The patient had pulmonary edema and uremic symptoms (vomiting and consciousness disorder). We performed brain MRI and electroencephalogram (EEG) to evaluate cerebral function.

**Figure 1:**
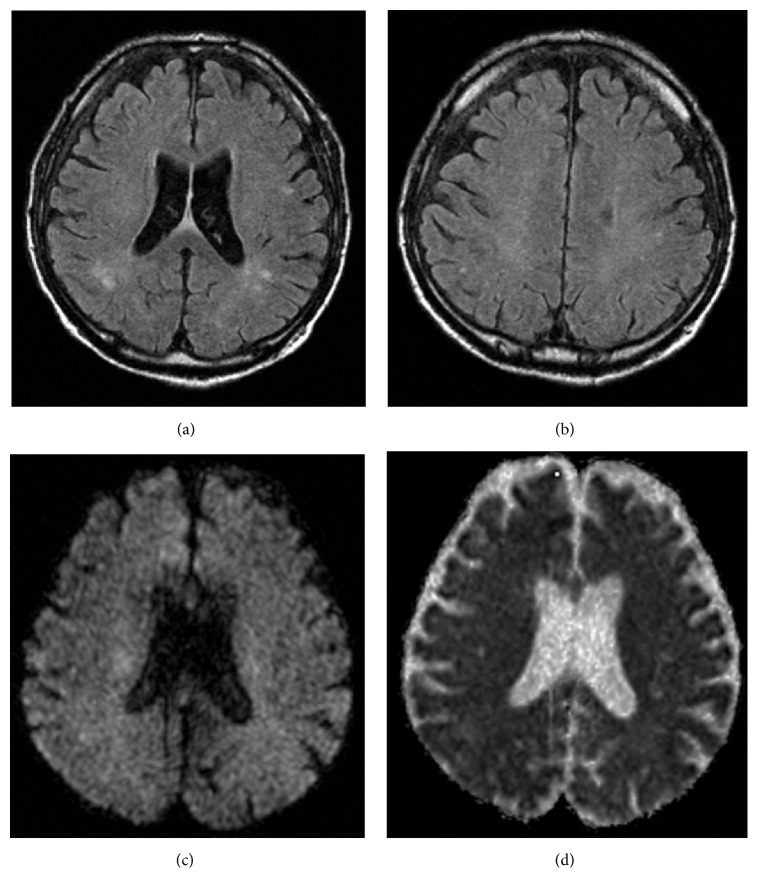
Cranial MRI on admission. ((a), (b)) Cranial T2-weighted fluid attenuated inversion recovery (FLAIR) MRI showing several old small brain infarctions. (c) Diffusion-weighted MR imaging (DWI) obtained on admission, showing no hyperintense signal alterations. (d) Apparent diffusion coefficient (ADC) mapping obtained on admission, showing no increased diffusion in cerebral lesions.

**Figure 2:**
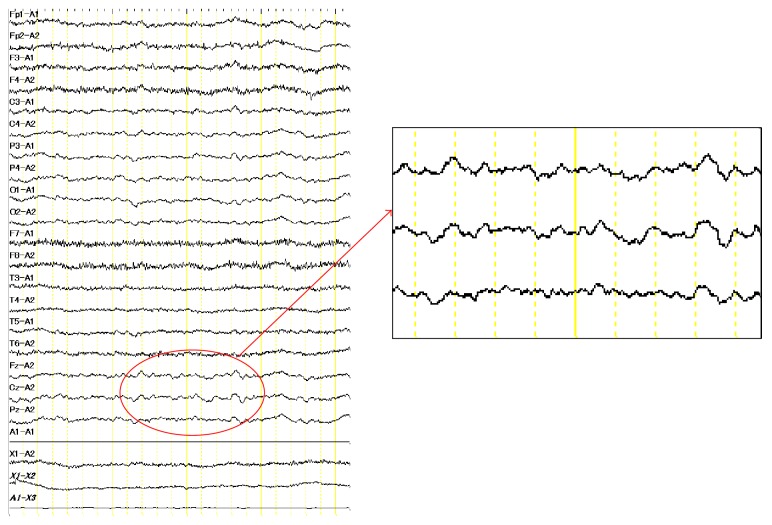
Electroencephalogram taken on admission, showing frequent slow waves (circle).

T2-weighted fluid attenuated inversion recovery (FLAIR) MRI showed several old, small brain infarcts (Figures 1(a) and 1(b)). Diffusion-weighted MR imaging (DWI) did not show increased apparent diffusion coefficient (ADC), which would be suggestive of vasogenic edema. EEG showed frequent slow waves, consistent with metabolic encephalopathy (Figure 2).

**Figure 3:**
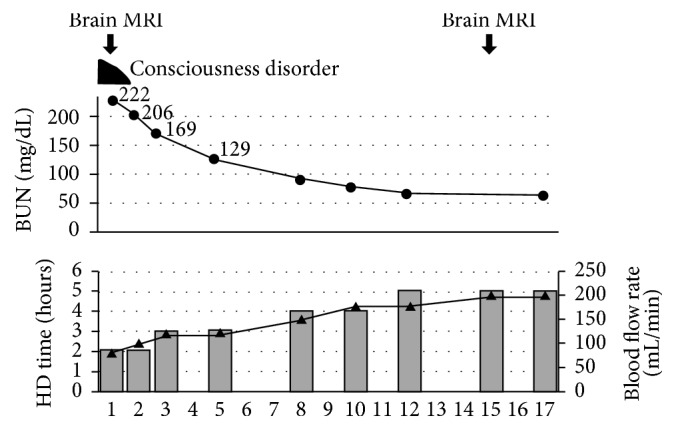
Clinical course during patient's initiation period. HD, hemodialysis; BUN, blood urea nitrogen; MRI, magnetic resonance imaging. Columns show changes in HD duration. Closed triangles show changes in HD blood flow rate.

The patient was placed on short-duration HD (2 hours) with smaller surface area (cellulose triacetate; membrane area: 0.7 m^2^) and low blood flow (100 mL/min) to avoid DDS (Figure 3). His consciousness gradually improved and he did not develop symptoms of DDS.

**Figure 4:**
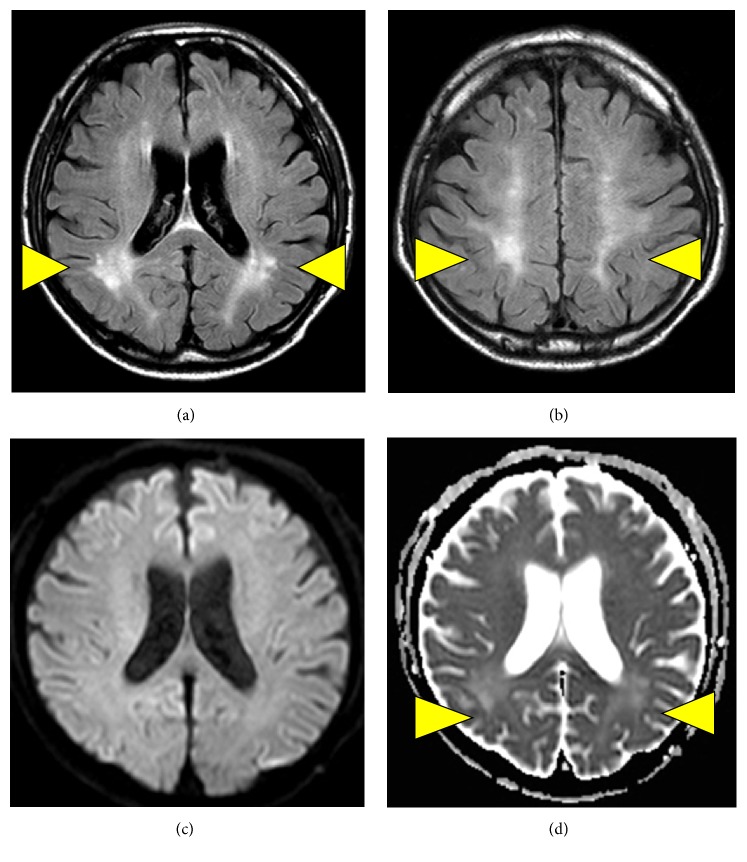
Cranial MRI taken on day 15, showing brain edema. ((a), (b)) T2-weighted FLAIR MRI showing increased signal intensities bilaterally in the cortical and subcortical areas of the occipital lobe (arrowhead). (c) DWI demonstrates no hyperintense signal alterations in these regions. (d) ADC is elevated in bilateral occipital lesions (arrowhead).

**Figure 5:**
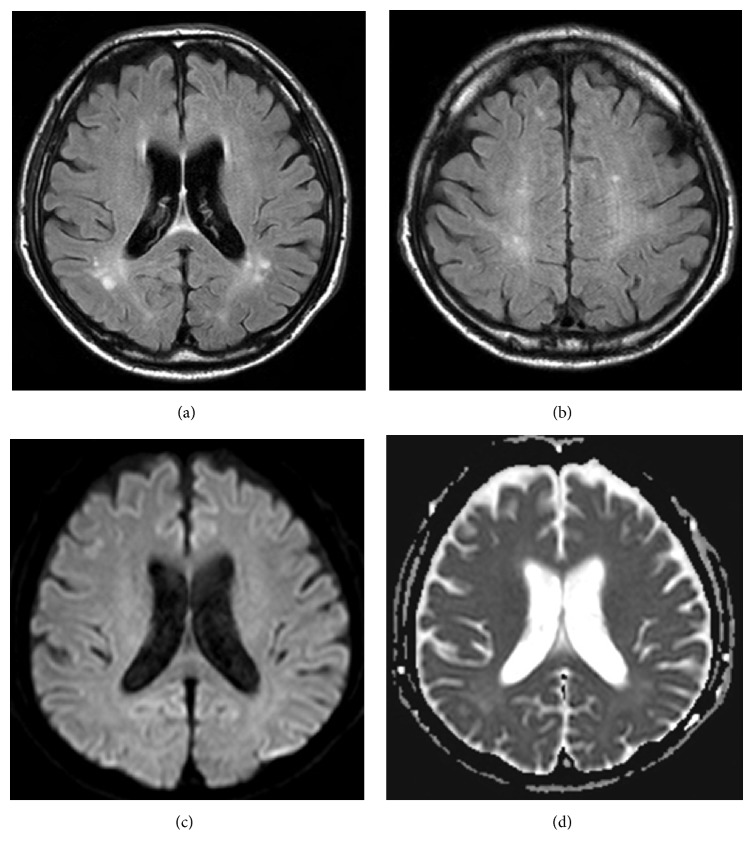
Brain MRI taken on day 29. ((a), (b)) Cranial T2-weighted FLAIR MRI showing several old small brain infarctions. (c) DWI obtained on day 29, showing no hyperintense signal alterations. (d) ADC mapping obtained on day 29, showing no increased diffusion in cerebral lesions.

On day 15, we repeated MRI and EEG. T2-weighted FLAIR MRI showed increased signal intensities bilaterally in the cortical and subcortical areas of the occipital lobe (Figures 4(a) and 4(b)). DWI demonstrated no hyperintense signal alterations in these regions. He had no cerebral infarcts, but the ADC was elevated in bilateral occipital lesions (Figures 4(c) and 4(d)). Thus, although the patient did not have clinical symptoms of DDS, we diagnosed that he had asymptomatic brain edema. EEG on day 15 had been compared with the findings on admission; namely, the slow waves on EEG had decreased, consistent with improving uremic encephalopathy. The patient underwent vascular access surgery for HD and continued maintenance HD. On day 29, a third MRI was performed and showed return to findings on admission (Figure 5).

The patient was discharged on regular intermittent HD without any neurological deficits. No further neurological disturbances were noted during 1-year follow-up.

## 3. Discussion

Uremic encephalopathy occurs in patients with renal insufficiency [8]. Various metabolic complications associated with uremia, including changes in levels of neurotransmitters, parathyroid hormone, calcium, acidosis, brain osmolality, cerebral blood flow, and amino acids, are believed to cause neurological manifestations in ESKD [9, 10].

Our patient had severe renal insufficiency and uremia. The uremic symptoms included confusion, which responded to dialysis. Our patient's blood pressure was not elevated and his brain MRI was normal on admission, and EEG showed frequent slow waves. Based on these results, we diagnosed the patient's neurological condition as uremic encephalopathy. Although we performed HD using a small surface area dialyzer with low blood flow rate for a short time of 2 hours to avoid DDS, T2-weighted FLAIR MRI obtained on the 15th day showed increased signal intensities bilaterally in the cortical and subcortical areas of the occipital lobe. These MRI changes are consistent with brain edema and with the clinical observation of DDS. Importantly, there were no radiological signs of cerebral or subarachnoid hemorrhage or thrombosis in sinus vein. The patient had no history of any neurological disturbances or seizures and no family history of neurological abnormalities. Although the patient's consciousness improved after HD initiation, cerebral edema appeared on MRI.

Cerebral edema is responsible for most of the manifestations of DDS. Cerebral edema in patients with DDS has been described as both cytotoxic edema and interstitial edema [2]. Chen et al. [11] reported that ESKD patients with severe azotemia had greater ADC than healthy subjects, a finding that worsened with HD. ADC measured by DWI is sensitive for detecting dynamic changes in tissue water content. The authors suggested that severe azotemia in ESKD leads to interstitial cerebral edema, reflected as increased ADC, and that further increases in ADC, reflecting edema associated with HD, are interstitial rather than cytotoxic in nature [11]. This situation is different from that of our case, but a similar mechanism can be considered in our case.

Actually, we selected short-duration HD (2 hours), smaller surface dialysis membrane, and low blood flow in initiation of dialysis for our patient. Therefore, BUN slowly declined (Figure 3). However, our patient had asymptomatic brain edema in initiation of hemodialysis. There are few reports on asymptomatic brain edema so far. In particular, there is no report on asymptomatic brain edema in DDS at all. In this regard, our case is very valuable and worth verifying.

Reversible posterior leukoencephalopathy syndrome (RPLS) is characterized by headache, seizures, and visual disturbance, often associated with an abrupt increase in blood pressure [12]. MRI findings of RPLS predominantly involve the posterior regions of the cerebral hemispheres and affect both gray and white matter. However, sustained rather than abrupt hypertension, absence of visual symptoms, and lack of characteristic imaging findings in our patient did not support the diagnosis of RPLS.

We have reported previously a patient with severe renal insufficiency who demonstrated RPLS with uremic encephalopathy without severe hypertension [13]. Other studies have reported that uremic encephalopathy can be associated with derangements in vascular autoregulatory mechanisms [14, 15]. One study reported that uremic toxin-associated dysfunction of the blood-brain barrier may account for the neurological findings in patients with ESKD before their first HD [15]. de Groot et al. [16] reported that uremia impairs endothelial function and inhibits differentiation of endothelial progenitor cells. This effect on the endothelium may explain the abnormal vascular autoregulatory mechanisms in the brain.

Although our patient had severe uremic symptoms, his blood pressure and respiratory condition were stable. Therefore, we did not pursue continuous hemofiltration or continuous hemodiafiltration. The patient did not have apparent DDS symptoms. However, the use of continuous hemofiltration or hemodiafiltration might have been advisable. In our patient, cerebral edema on MRI had regressed within approximately a month. When we begin HD in ESKD patients with severe uremia, we should be aware of the possibility of cerebral edema, even in the absence of apparent symptoms. No previous report investigated data about dialysis induced asymptomatic brain edema.

In summary, we have described an ESKD patient with uremic encephalopathy who had serial changes on MRI and EEG after HD initiation. Our patient's radiological findings were similar to those described in patients with cerebral edema resulting from DDS. MRI findings in ESKD patients without DDS symptoms help to clarify the diagnosis of cerebral edema. In this case, the patient did not have apparent DDS symptoms and was therefore diagnosed with asymptomatic cerebral edema.
